# Incidence, survival, and prognostic factors for patients with gastrointestinal mixed neuroendocrine non-neuroendocrine neoplasms: a SEER population-based study

**DOI:** 10.1007/s00432-023-05356-z

**Published:** 2023-09-01

**Authors:** Boqi Xu, Fan Zhang, Runda Wu, Yao Peng, Zhongqi Mao, Shan Tong

**Affiliations:** Department of General Surgery, The First Affiliated Hospital of Soochow University, Suzhou, China

**Keywords:** Mixed neuroendocrine non-neuroendocrine neoplasms, Incidence, Survival, Prognostic factors, Nomogram

## Abstract

**Background:**

Mixed neuroendocrine non-neuroendocrine neoplasms (MiNENs) are a group of rare tumors with limited research currently available. This study aimed to analyze the incidence, survival, and prognostic factors of gastrointestinal MiNENs.

**Methods:**

We included data from the Surveillance, Epidemiology, and End Results (SEER) database between 2000 and 2019. We compared the clinicopathologic characteristics and survival rates between MiNENs and neuroendocrine tumors (NETs), and calculated the incidence of MiNENs. We utilized univariate and multivariate Cox analysis to assess independent factors of prognosis and established a nomogram to predict 1-, 2-, and 3-year cancer-specific survival (CSS). Calibration and receiver operating characteristic (ROC) curves were drawn to validate the accuracy and reliability of the model. Decision curve analysis (DCA) was used to assess the clinical utility of the model.

**Results:**

Patients with gastrointestinal MiNENs had a poorer prognosis than those with NETs. The overall incidence of gastrointestinal MiNENs has been increasing annually. Multivariate Cox regression analysis revealed that tumor size, lymph node metastasis, distant metastasis, and surgery were independent risk factors for CSS in MiNENs patients. Based on these risk factors, the 1-, 2-, and 3-year CSS nomogram model for MiNENs patients was established. Calibration, ROC, and DCA curves of the training and validation sets demonstrated that this model had good accuracy and clinical utility.

**Conclusion:**

Gastrointestinal MiNENs are rare tumors with an increasing incidence rate. The nomogram model is expected to be an effective tool for personalized prognosis prediction in MiNENs patients, which may benefit clinical decision-making.

## Introduction

Mixed neuroendocrine non-neuroendocrine neoplasms (MiNENs) are a group of rare and heterogeneous tumors (Modlin et al. 2008). In the 2010 WHO classification, this subtype was known as mixed adeno-neuroendocrine carcinomas (MANECs), presenting features of both adenocarcinoma and neuroendocrine carcinoma (Mestier et al. 2017; Huang et al. 2021). As per the updated 2019 classification, the non-neuroendocrine components of the tumor include adenoma, adenocarcinoma, squamous cell carcinoma, and acinar cell carcinoma, while the neuroendocrine component comprises well-differentiated and poorly differentiated neuroendocrine neoplasms (Jiang et al. 2023; Nagtegaal et al. 2020). Neuroendocrine and non-neuroendocrine components each constitute 30% or more of tumors (Assarzadegan and Montgomery 2021). To better incorporate the heterogeneous nature of these mixed tumors, the term “MiNENs” has now been adopted.

Despite the discovery of MiNENs in various organs such as the stomach, intestine, pancreas, biliary tract, appendix, and cervix, there remains a scarcity of comprehensive research on the topic due to the novelty of this concept (Huang et al. 2021; Oneda et al. 2019). As a result, most current researches are limited to only a small number of case reports and retrospective studies with inadequate sample sizes (Jiang et al. 2023; Oneda et al. 2019; Iwasaki et al. 2022). Hence, the incidence, clinical characteristics, and prognosis of MiNENs have yet to be fully understood.

The objective of this research was to distinguish the clinical and pathological characteristics and survival rates of gastrointestinal MiNENs and neuroendocrine tumors (NETs) by analyzing data obtained from the Surveillance, Epidemiology, and End Results (SEER) database. This study aimed to investigate and analyze the incidence and prognostic factors of gastrointestinal MiNENs. Furthermore, a predictive nomogram was developed to forecast the survival rates of patients with MiNENs, addressing the limitations of retrospective studies and providing precise predictions of survival outcomes.

## Methods and materials

### Data source

Cases of gastrointestinal MiNENs and NETs were identified from the SEER database. The SEER database, which comes from cancer registries in 19 regions of the United States, represents about 35% of the population (https://www.cancer.gov/research/areas/public-health/what-is-seer-infographic). We used the SEER database version available on April 2022 (November 2021 Submission).

### Patients and data collection

Patient data of gastrointestinal MiNENs and NETs from 2000 to 2019 were obtained from the SEER database according to the International Classification of Diseases for Oncology, the Third Edition (ICD-O-3, the site codes: stomach C16.0–16.9, small intestine C17.0–17.9, appendix C18.1, colon C18.0 and 18.2–18.9, rectum C19.9 and 20.9) (Cai et al. 2022).

The following inclusion criteria were used: (1) tumor histological type: MiNENs (SEER histology code: 8244/3)(Song et al. 2022), and NETs (SEER histology codes: 8013/3, 8041/3, 8150/3–8157/3, 8240/3, 8241/3, 8242/3, 8243/3, 8246/3, 8249/3) (Cai et al. 2022; Shah et al. 2019; Miao et al. 2018); (2) confirmation of MiNENs and NETs diagnosis histologically or microscopically. The following were the exclusion criteria: (1) survival time was unknown; (2) incomplete clinical and pathological data.

Patient clinical and pathological variables included age, gender, race, marital status, tumor site, tumor grade, tumor size, TNM staging, regional nodes examined, and therapies employed (primary tumor surgery, metastasectomy, and chemotherapy).

The diagnosis year was determined based on the SEER code “YEAR OF DIAGNOSIS,” which referred to the year when the tumor was first diagnosed by a recognized medical practitioner, regardless of clinical or microscopic confirmation. The tumor size and regional nodes examined were determined based on the SEER codes “CS TUMOR SIZE” and “REGIONAL NODES EXAMINED (1988 +)”. These variables were transformed into categorical variables using the median values of 2 and 9 as cutoff points. The SEER code “GRADE (THRU 2017)” determined the tumor grade and followed the International Classification of Diseases for Oncology, the Second Edition (ICD-O-2) classification system, which categorized tumors into four grades.

### Statistical analysis

The R version 4.2.2 (http://www.R-project.org/) was employed for all statistical analyses. All patients who were still alive at the time of analysis were included in the study. Descriptive statistics for categorical variables were analyzed using proportions and the Chi-square test of independence. We analyzed the overall incidence rates of MiNENs from 2000 to 2019 and further demonstrated the differences in incidence rates among different ages, genders, and tumor sites. To estimate the extent of changes in incidence rates, we utilized annual percent change (APC) and 95% confidence interval (CI). The primary endpoints were identified as the overall survival (OS) and cancer-specific survival (CSS) rates. The OS rate was defined as the duration between the diagnosis of MiNENs or NETs and death from any cause. The CSS rate was the period between the diagnosis of MiNENs or NETs and death directly caused by the same cancer. Kaplan–Meier curves and log-rank tests were employed to generate OS and CSS curves for different patient subgroups.

After excluding MiNENs patients who did not die from cancer-specific causes, a random sampling method was employed to divide the remaining MiNENs patients into a training set and a validation set in a ratio of 7:3. In the training set, univariate Cox regression was used to identify relevant prognostic factors with a P-value less than 0.05, followed by multivariate Cox regression analysis to determine the independent prognostic factors and record the hazard ratio (HR) and 95% CI.

Based on the independent prognostic factors, a nomogram using the “rms” package in R software was constructed to predict the 1-, 2-, and 3-year CSS of patients with gastrointestinal MiNENs. The weight of each factor in the nomogram was determined by the regression coefficient in the Cox regression analysis (Iasonos et al. 2008). The accuracy of the nomogram was validated by calibration curves in both the training and validation sets, and the discriminatory ability was evaluated by drawing receiver operating characteristic (ROC) curves and calculating the area under the curve (AUC). Finally, decision curve analysis (DCA) was performed to assess the potential clinical value of the nomogram.

## Results

### Patient characteristics

**Fig. 1:**
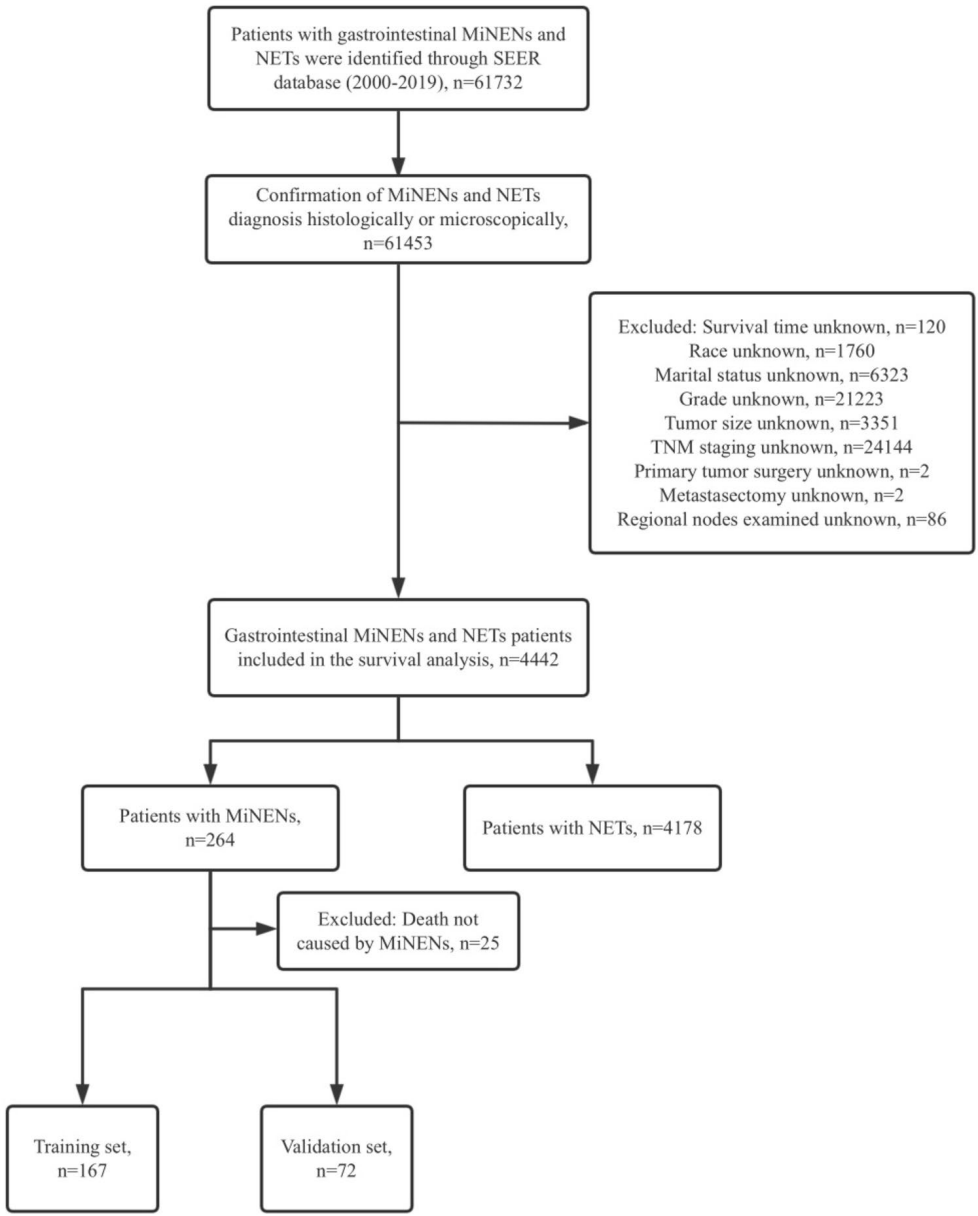
Inclusion and exclusion procedures for MiNENs patients from SEER database. *SEER* Surveillance, Epidemiology, and End Results, *MiNENs* mixed neuroendocrine non-neuroendocrine neoplasms, *NETs* neuroendocrine tumors

The SEER database initially provided data on 61,732 patients between 2000 and 2019. After screening, 4442 patients were included in the final survival prognosis analysis, of which 264 were MiNENs (5.9%) and 4178 were NETs (94.1%). Figure 1 illustrated the data selection process. The patient characteristics were shown in Table 1.

**Table 1:** Clinical and pathological characteristics of MiNENs and NETs Patients

	MiNENs (N = 264),* n*%	NETs (N = 4178),* n*%	Overall (N = 4442),* n*%	*p*
Age				0.869
< 60 years	109 (41.3%)	1755 (42.0%)	1864 (42.0%)	
≥ 60 years	155 (58.7%)	2423 (58.0%)	2578 (58.0%)	
Sex				0.827
Female	135 (51.1%)	2099 (50.2%)	2234 (50.3%)	
Male	129 (48.9%)	2079 (49.8%)	2208 (49.7%)	
Race				0.826
AI/AN	1 (0.4%)	17 (0.4%)	18 (0.4%)	
API	16 (6.1%)	213 (5.1%)	229 (5.2%)	
Black	32 (12.1%)	571 (13.7%)	603 (13.6%)	
White	215 (81.4%)	3377 (80.8%)	3592 (80.9%)	
Marital status				0.955
Married	159 (60.2%)	2532 (60.6%)	2691 (60.6%)	
Single	105 (39.8%)	1646 (39.4%)	1751 (39.4%)	
Primary tumor site				< 0.001
Stomach	16 (6.1%)	465 (11.1%)	481 (10.8%)	
Small intestine	13 (4.9%)	1762 (42.2%)	1775 (40.0%)	
Appendix	134 (50.8%)	307 (7.3%)	441 (9.9%)	
Colon	88 (33.3%)	1001 (24.0%)	1089 (24.5%)	
Rectum	13 (4.9%)	643 (15.4%)	656 (14.8%)	
Grade				< 0.001
I	35 (13.3%)	2285 (54.7%)	2320 (52.2%)	
II	58 (22.0%)	609 (14.6%)	667 (15.0%)	
III	138 (52.3%)	869 (20.8%)	1007 (22.7%)	
IV	33 (12.5%)	415 (9.9%)	448 (10.1%)	
Tumor size				< 0.001
> 2 cm	199 (75.4%)	2201 (52.7%)	2400 (54.0%)	
≤ 2 cm	65 (24.6%)	1977 (47.3%)	2042 (46.0%)	
T stage				< 0.001
T0–2	39 (14.8%)	1687 (40.4%)	1726 (38.9%)	
T3–4	225 (85.2%)	2491 (59.6%)	2716 (61.1%)	
N stage				< 0.001
N0	111 (42.0%)	1767 (42.3%)	1878 (42.3%)	
N1	77 (29.2%)	1784 (42.7%)	1861 (41.9%)	
N2–3	76 (28.8%)	627 (15.0%)	703 (15.8%)	
M stage				0.815
M0	183 (69.3%)	2933 (70.2%)	3116 (70.1%)	
M1	81 (30.7%)	1245 (29.8%)	1326 (29.9%)	
Primary tumor surgery				< 0.001
No surgery	7 (2.7%)	355 (8.5%)	362 (8.1%)	
Local resection	71 (26.9%)	2146 (51.4%)	2217 (49.9%)	
Radical resection	186 (70.5%)	1677 (40.1%)	1863 (41.9%)	
Metastasectomy				0.019
No	220 (83.3%)	3692 (88.4%)	3912 (88.1%)	
Yes	44 (16.7%)	486 (11.6%)	530 (11.9%)	
Chemotherapy				< 0.001
No	123 (46.6%)	3328 (79.7%)	3451 (77.7%)	
Yes	141 (53.4%)	850 (20.3%)	991 (22.3%)	
Regional nodes examined				< 0.001
< 9	61 (23.1%)	2146 (51.4%)	2207 (49.7%)	
≥ 9	203 (76.9%)	2032 (48.6%)	2235 (50.3%)	

*MiNENs* mixed neuroendocrine non-neuroendocrine neoplasms, *NETs* neuroendocrine tumors, *AI/AN* American Indian/Alaska Native, *API* Asian or Pacific Islander

Among these patients, there were significant differences in the primary tumor site (*P* < 0.001) and tumor grade (*P* < 0.001). MiNENs were commonly found in the appendix (50.8%) and mostly classified as grade III (52.3%). On the other hand, NETs were more frequently observed in the small intestine (42.2%) and mainly classified as grade I (54.7%).

Additionally, compared with NETs patients, patients with MiNENs were more likely to have a larger tumor size (75.4% vs. 52.7%, *P* < 0.001), T3–4 stage (85.2% vs. 59.6%, *P* < 0.001), and N2–3 stage (28.8% vs. 15.0%, *P* < 0.001).

In terms of surgical treatment, MiNENs patients had a greater tendency to elect for radical resection (70.5%), while NENs patients tended to choose local resection more often (51.4%) (*P* < 0.001). Compared to NETs patients, a greater proportion of MiNENs patients chose to undergo metastasectomy (16.7% vs. 11.6%, *P* = 0.019) and chemotherapy (53.4% vs. 20.3%, *P* < 0.001). Additional cohort information was shown in Table 1.

### Incidence and trend of MiNENs

**Fig. 2:**
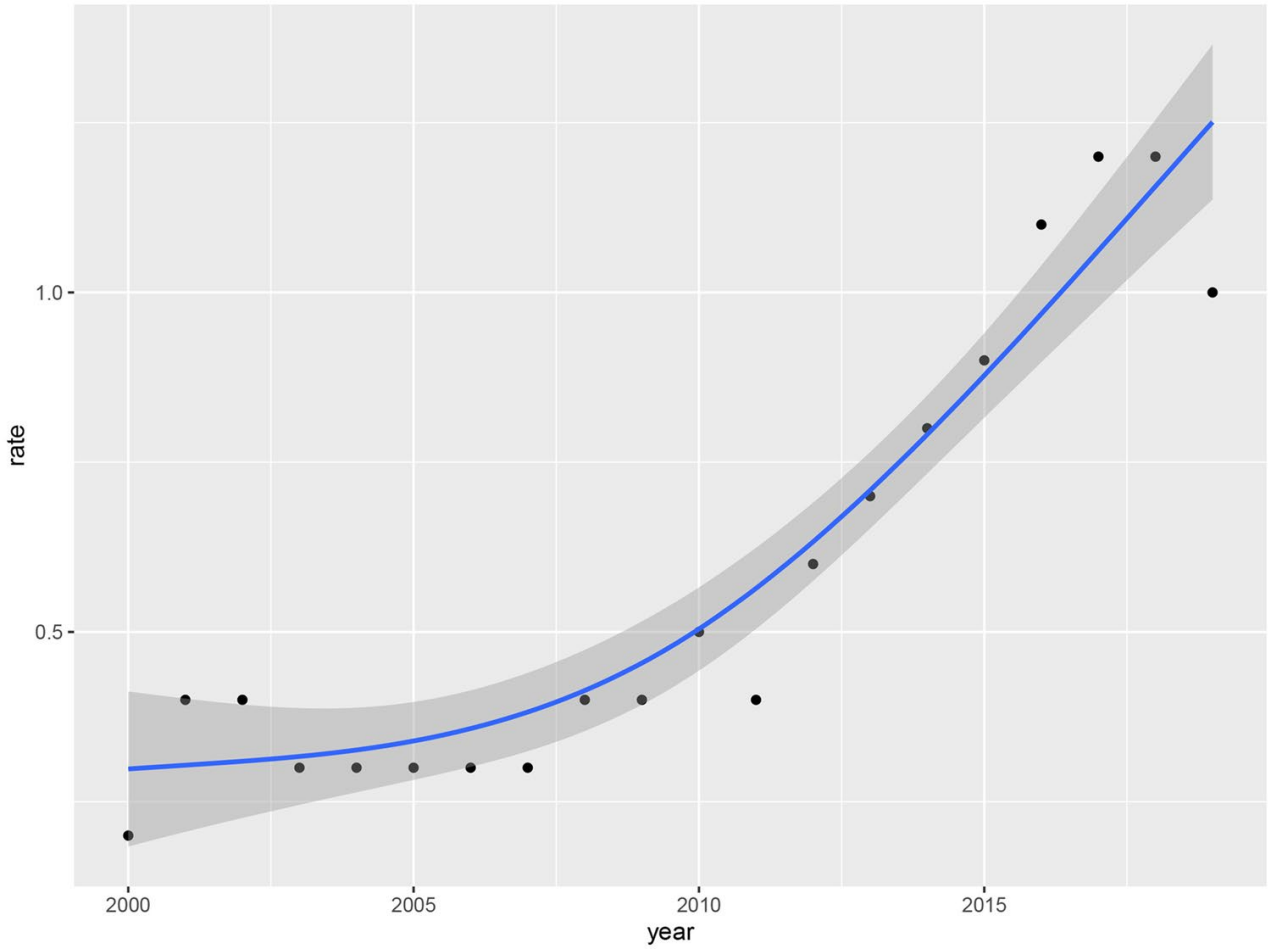
Incidence and trend of MiNENs between 2000 and 2019 (per 1,000,000 person-years). *MiNENs* mixed neuroendocrine non-neuroendocrine neoplasms

**Fig. 3:**
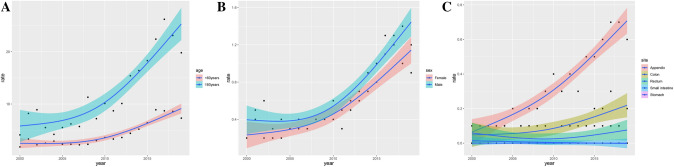
Incidence and trends of different subgroups of MiNENs patients. **a** Incidence and trends of MiNENs patients of different ages; **b** incidence and trends of MiNENs patients of different genders; **c** incidence and trends of MiNENs in different sites. *MiNENs* mixed neuroendocrine non-neuroendocrine neoplasms

As shown in Fig. 2, the incidence rate of MiNENs exhibited a significant upward trend for the entire population between 2000 and 2019 (APC = 9.293, 95% CI 7.525–11.090, *P* < 0.05), reaching its peak in 2017 (1.225 cases per 1,000,000 person-years). Figure 3 illustrated the variations in tumor incidence rates among patients of different ages, genders, and tumor sites. We have observed a higher incidence rate of MiNENs in males and individuals aged 60 and above. The occurrence of MiNENs in the appendix was significantly higher than in other sites (*P* < 0.05), with the peak incidence being observed in 2017 (0.735 cases per 1,000,000 person-years, 95% CI 0.575–0.928).

### Survival analysis between different pathological subgroups

**Fig. 4:**
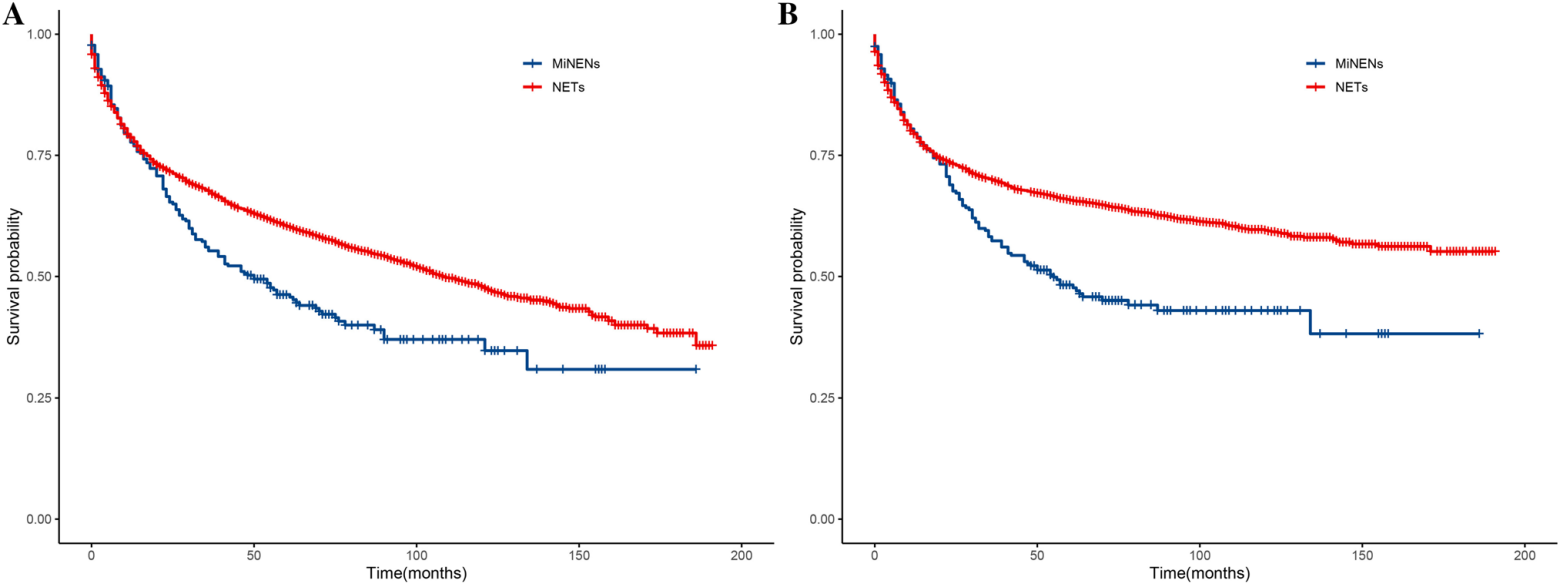
KM survival curves comparing the OS and CSS of patients in different pathological subgroups. **a** OS of patients with MiNENs and NETs; **b** CSS of patients with MiNENs and NETs. *KM* Kaplan–Meier, *OS* overall survival, *CSS* cancer-specific survival, *MiNENs* mixed neuroendocrine non-neuroendocrine neoplasms, *NETs* neuroendocrine tumors

In our study, we used Kaplan–Meier curves to examine the correlation between tumor pathology and clinical outcomes, including OS and CSS curves. As shown in Fig. 4, patients with MiNENs had lower OS and CSS rates than patients with NETs. The 1-, 2-, and 3-year OS and CSS rates were presented in Table 2. The 3-year CSS rate in NETs patients reached up to 0.700 (0.685–0.715), while in MiNENs patients, it was only 0.573 (0.514–0.640). Furthermore, we also calculated the median OS time for MiNENs and NETs patients, which were 50 months (36–70 months) and 108 months (102–120 months), respectively.

**Table 2:** 1-, 2-, 3-Year OS and CSS rates of patients with MiNENs and NETs

	MiNENs	NETs
1-Year OS rate (95%CI)	0.777 (0.728–0.829)	0.787 (0.775–0.799)
2-Year OS rate (95%CI)	0.653 (0.598–0.714)	0.716 (0.703–0.730)
3-Year OS rate (95%CI)	0.553 (0.496–0.617)	0.676 (0.662–0.691)
1-Year CSS rate (95%CI)	0.796 (0.746–0.849)	0.794 (0.781–0.808)
2-Year CSS rate (95%CI)	0.676 (0.619–0.739)	0.732 (0.718–0.747)
3-Year CSS rate (95%CI)	0.573 (0.514–0.640)	0.700 (0.685–0.715)

*OS* overall survival, *CSS* cancer-specific survival, *MiNENs* mixed neuroendocrine non-neuroendocrine neoplasms, *NETs* neuroendocrine tumors, *CI* confidence interval

### Feature selection and nomogram construction

A total of 239 MiNENs patients were randomly divided into a training set (*N* = 167) and a validation set (*N* = 72) with a 7:3 ratio. Table 3 displayed their clinical and pathological characteristics. There were no significant differences in the clinical and pathological features between the two groups of patients.

**Table 3:** Baseline clinical characteristics of MiNENs patients in the training and validation sets

	Training (N = 167)	Validation (N = 72)	Overall (N = 239)	*χ*^*2*^	*p*
Age				0.02	0.893
< 60 years	71 (42.5%)	32 (44.4%)	103 (43.1%)		
≥ 60 years	96 (57.5%)	40 (55.6%)	136 (56.9%)		
Sex				0.01	0.943
Female	81 (48.5%)	36 (50.0%)	117 (49.0%)		
Male	86 (51.5%)	36 (50.0%)	122 (51.0%)		
Race				0.22	0.895
API	9 (5.4%)	5 (6.9%)	14 (5.9%)		
Black	21 (12.6%)	9 (12.5%)	30 (12.6%)		
White	137 (82.0%)	58 (80.6%)	195 (81.6%)		
Marital status				3.45	0.063
Married	111 (66.5%)	38 (52.8%)	149 (62.3%)		
Single	56 (33.5%)	34 (47.2%)	90 (37.7%)		
Primary tumor site				8.53	0.074
Stomach	11 (6.6%)	3 (4.2%)	14 (5.9%)		
Small intestine	5 (3.0%)	7 (9.7%)	12 (5.0%)		
Appendix	84 (50.3%)	38 (52.8%)	122 (51.0%)		
Colon	60 (35.9%)	18 (25.0%)	78 (32.6%)		
Rectum	7 (4.2%)	6 (8.3%)	13 (5.4%)		
Grade				6.90	0.075
I	20 (12.0%)	10 (13.9%)	30 (12.6%)		
II	44 (26.3%)	8 (11.1%)	52 (21.8%)		
III	83 (49.7%)	43 (59.7%)	126 (52.7%)		
IV	20 (12.0%)	11 (15.3%)	31 (13.0%)		
Tumor size				0.06	0.812
> 2 cm	127 (76.0%)	53 (73.6%)	180 (75.3%)		
≤ 2 cm	40 (24.0%)	19 (26.4%)	59 (24.7%)		
T stage				0.09	0.764
T0–2	25 (15.0%)	9 (12.5%)	34 (14.2%)		
T3–4	142 (85.0%)	63 (87.5%)	205 (85.8%)		
N stage				1.73	0.420
N0	75 (44.9%)	26 (36.1%)	101 (42.3%)		
N1	45 (26.9%)	24 (33.3%)	69 (28.9%)		
N2–3	47 (28.1%)	22 (30.6%)	69 (28.9%)		
M stage				1.19	0.275
M0	118 (70.7%)	45 (62.5%)	163 (68.2%)		
M1	49 (29.3%)	27 (37.5%)	76 (31.8%)		
Primary tumor surgery				0.09	0.955
No surgery	5 (3.0%)	2 (2.8%)	7 (2.9%)		
Local resection	47 (28.1%)	19 (26.4%)	66 (27.6%)		
Radical resection	115 (68.9%)	51 (70.8%)	166 (69.5%)		
Metastasectomy				0.86	0.355
No	142 (85.0%)	57 (79.2%)	199 (83.3%)		
Yes	25 (15.0%)	15 (20.8%)	40 (16.7%)		
Chemotherapy				0.03	0.872
No	73 (43.7%)	33 (45.8%)	106 (44.4%)		
Yes	94 (56.3%)	39 (54.2%)	133 (55.6%)		
Regional nodes examined				2.73	0.098
< 9	35 (21.0%)	23 (31.9%)	58 (24.3%)		
≥ 9	132 (79.0%)	49 (68.1%)	181 (75.7%)		

*MiNENs* mixed neuroendocrine non-neuroendocrine neoplasms, *NETs* neuroendocrine tumors, *API* Asian or Pacific Islander

To investigate the risk factors associated with the long-term survival outcome of MiNENs, univariate and multivariate Cox regression analyses were conducted to identify protective or adverse prognostic factors in the training set. The results of the multivariate Cox regression analysis, as presented in Table 4, indicated that among patients with MiNENs, tumors size less than 2 cm (HR 0.45, 95% CI 0.25–0.82, P = 0.027), local resection (HR 0.16, 95% CI 0.06–0.40, P = 0.001), and radical resection (HR 0.28, 95% CI 0.12–0.65, P = 0.013) were identified as protective prognostic factors. On the other hand, N1 stage (HR 2.02, 95% CI 1.21–3.36, P = 0.023), N2–3 stage (HR 4.18, 95% CI 2.55–6.85, P < 0.001), and M1 stage (HR 2.86, 95% CI 1.92–4.26, P < 0.001) were identified as adverse prognostic factors.

**Table 4:** Univariate and multivariate analyses for MiNENs Patients

Characteristics	Univariable			Multivariable		
	HR	95%CI	*p*	HR	95%CI	*p*
Age						
< 60 years	Reference					
≥ 60 years	1.55	1.07–2.25	0.054			
Sex						
Female	Reference					
Male	0.86	0.60–1.23	0.495			
Race						
API	Reference					
Black	0.98	0.32–2.98	0.974			
White	1.52	0.58–4.00	0.479			
Marital status						
Married	Reference					
Single	0.90	0.61–1.34	0.669			
Primary tumor site						
Stomach	Reference					
Small intestine	1.49	0.51–4.31	0.539			
Appendix	0.49	0.24–1.03	0.113			
Colon	1.19	0.58–2.46	0.686			
Rectum	0.87	0.30–2.51	0.824			
Grade						
I	Reference			Reference		
II	3.15	1.28–7.72	0.035	1.50	0.59–3.82	0.472
III	3.73	1.58–8.80	0.012	1.43	0.58–3.52	0.519
IV	5.30	2.06–13.62	0.004	1.92	0.71–5.20	0.280
Tumor size						
> 2 cm	Reference			Reference		
≤ 2 cm	0.30	0.17–0.53	0.001	0.45	0.25–0.82	0.027
T stage						
T0–2	Reference					
T3–4	1.72	0.96–3.07	0.125			
N stage						
N0	Reference			Reference		
N1	2.59	1.58–4.25	0.002	2.02	1.21–3.36	0.023
N2–3	6.82	4.30–10.82	< 0.001	4.18	2.55–6.85	< 0.001
M stage						
M0	Reference			Reference		
M1	3.92	2.72–5.66	< 0.001	2.86	1.92–4.26	< 0.001
Primary tumor surgery						
No surgery	Reference			Reference		
Local resection	0.10	0.04–0.23	< 0.001	0.16	0.06–0.40	0.001
Radical resection	0.16	0.07–0.36	< 0.001	0.28	0.12–0.65	0.013
Metastasectomy						
No	Reference					
Yes	0.94	0.56–1.56	0.829			
Chemotherapy						
No	Reference					
Yes	1.47	1.01–2.15	0.092			
Regional nodes examined						
< 9	Reference					
≥ 9	0.75	0.49–1.16	0.277			

*MiNENs* mixed neuroendocrine non-neuroendocrine neoplasms, *HR* hazard ratio, *CI* confidence interval, *API* Asian or Pacific Islander

**Fig. 5:**
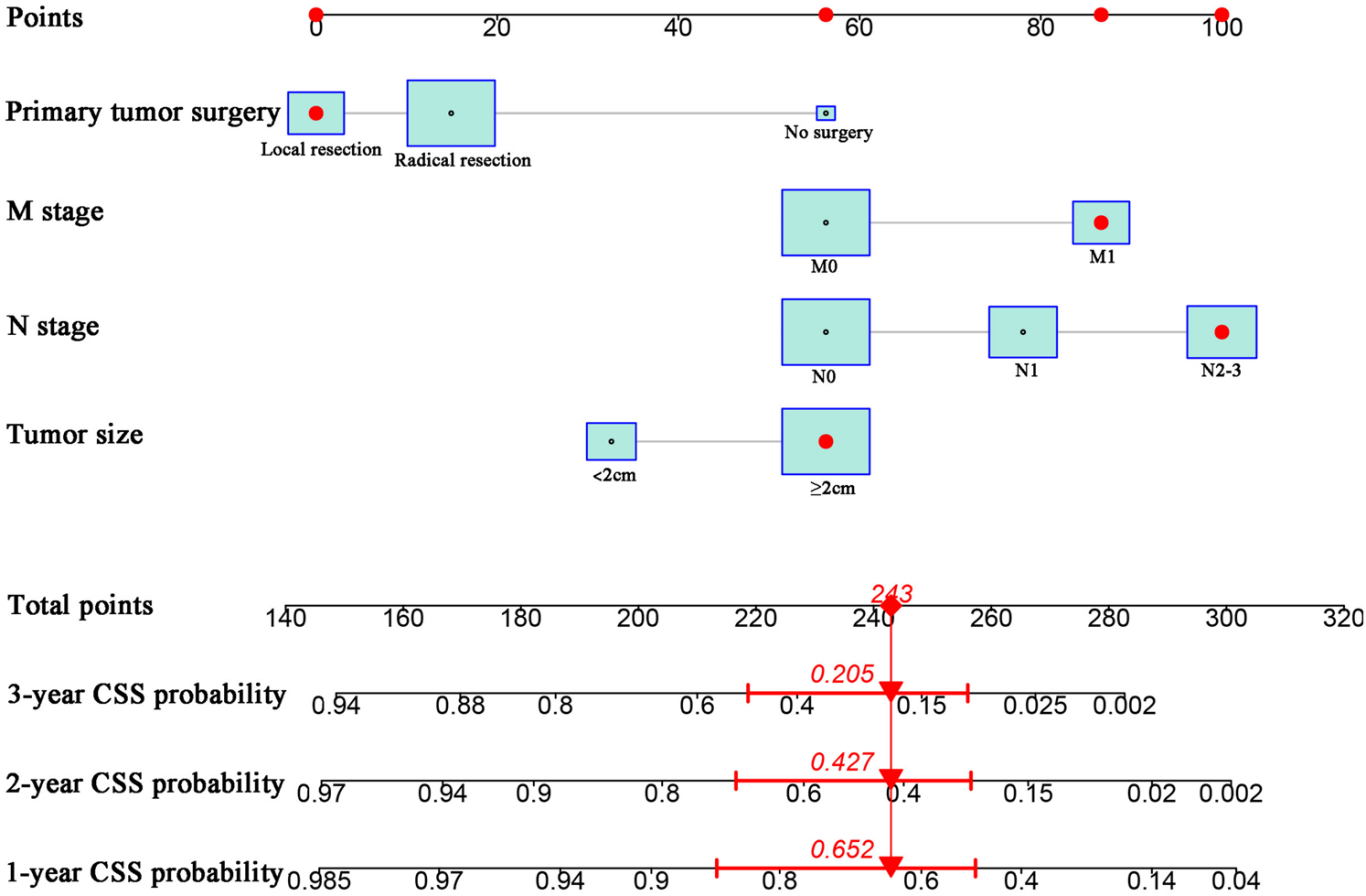
A nomogram for predicting the 1-, 2-, and 3- year CSS in patients with MiNENs. *CSS* cancer-specific survival, *MiNENs* mixed neuroendocrine non-neuroendocrine neoplasms

Based on the identified risk factors, we developed a nomogram model using the “rms” package in the R software to predict the 1-, 2-, and 3-year CSS rates of patients with gastrointestinal MiNENs (Fig. 5). The model assigned a specific score to each level of these variables on the scale. By summing the scores of each variable, the total score of each patient can be obtained. Subsequently, we can project the total score onto the total score scale of the nomogram to predict the probability of 1-, 2-, and 3-year CSS.

### Performance and validation of the nomogram

**Fig. 6:**
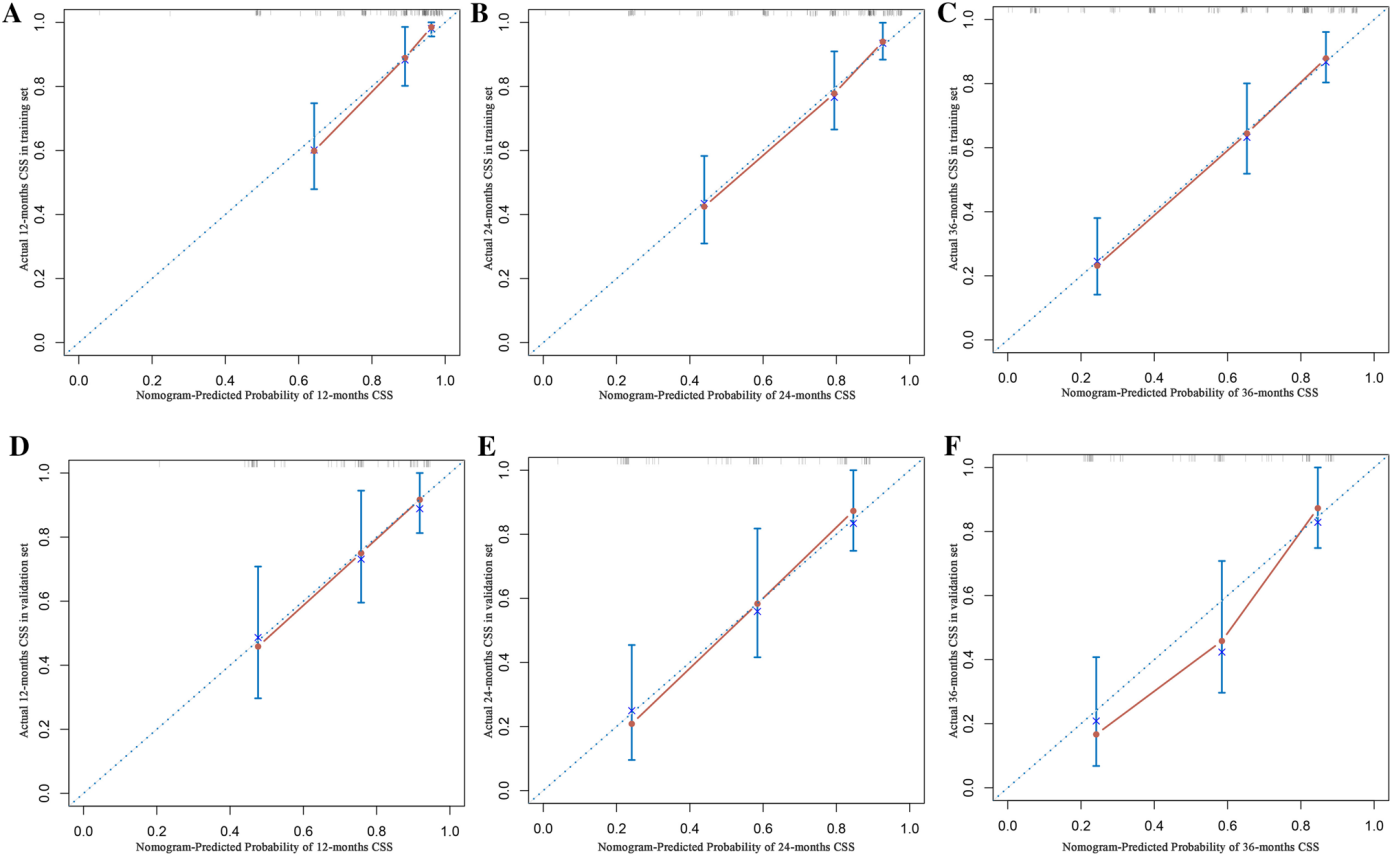
Calibration curves for evaluating the accuracy of the nomogram. **a**–**c** The calibration curves for predicting the 1-, 2-, 3-year CSS in the training set; **d**–**f** the calibration curves for predicting the 1-, 2-, 3-year CSS in the validation set. *CSS* cancer-specific survival

**Fig. 7:**
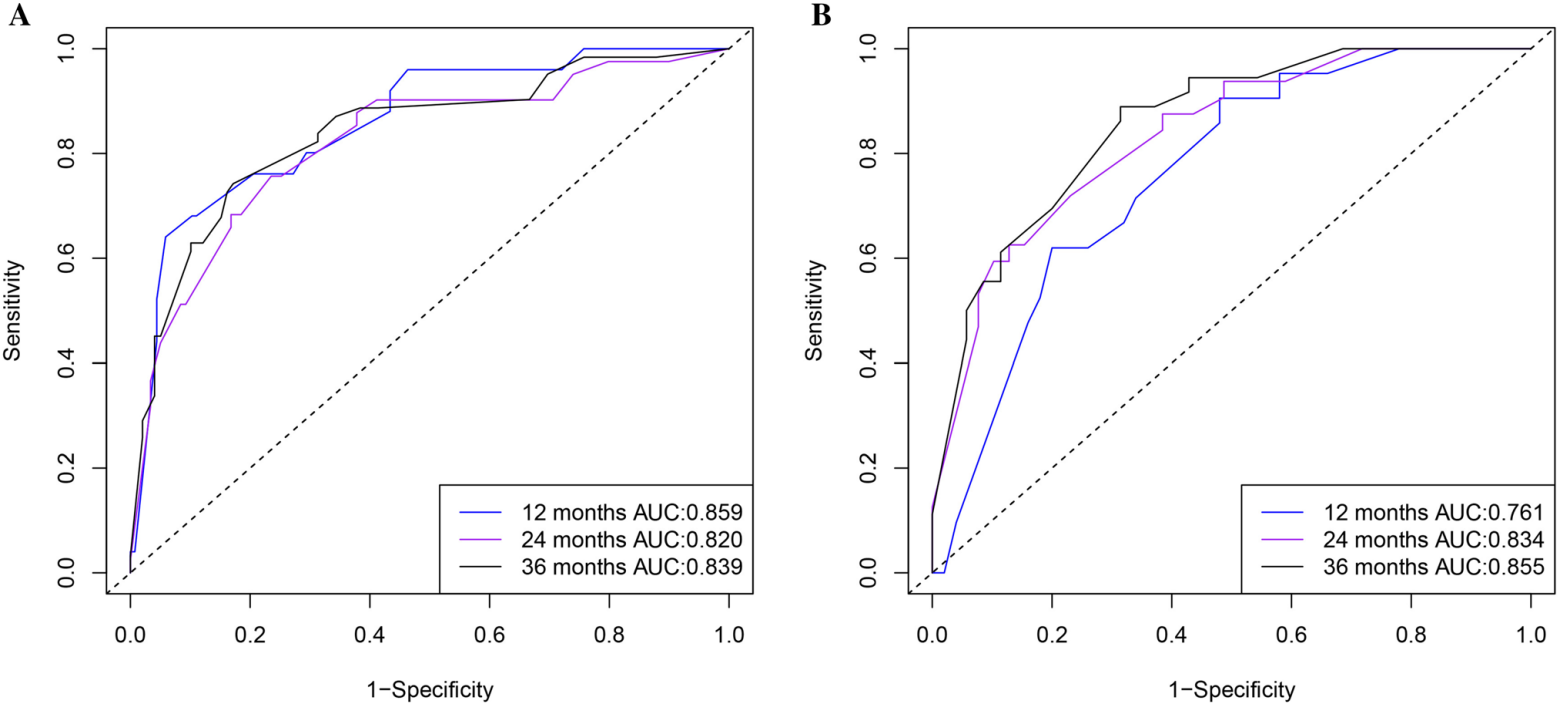
ROC curves for evaluating the discriminability of the nomogram. **a** ROC curves for the 1-, 2-, and 3- year CSS in the training set; **b** ROC curves for the 1-, 2-, and 3- year CSS in the validation set. *ROC* receiver operating characteristic, *AUC* area under the curve, *CSS*: cancer-specific survival

**Fig. 8:**
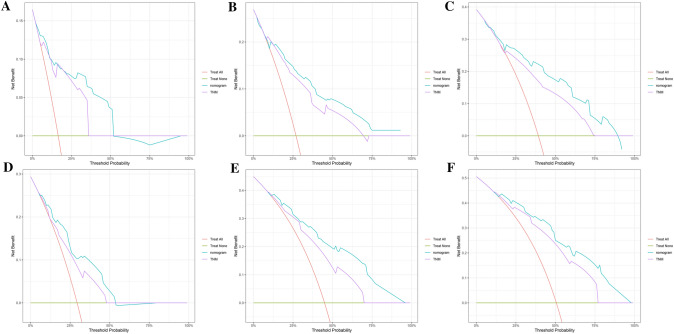
DCA curves for evaluating the potential clinical value of the nomogram. **a**–**c** DCA curves of the nomogram and AJCC TNM staging system for predicting the 1-, 2-, and 3-year CSS in the training set; **d**–**f** DCA curves of the nomogram and AJCC TNM staging system for predicting the 1-, 2-, and 3-year CSS in the validation set. *DCA* decision curve analysis, *CSS* cancer-specific survival

The calibration curves of the nomogram exhibited high consistency between the predicted and actual probabilities of CSS in the training and validation sets, indicating good accuracy of the model (Fig. 6). In the training set, the AUC values of the nomogram for 1-, 2-, 3- year CSS were 0.859 (95% CI 0.780–0.938), 0.820 (95% CI 0.741–0.899), and 0.839 (95% CI 0.774–0.904), respectively (Fig. 7a). In the validation set, the AUC values of the nomogram for 1-, 2-, 3- year CSS were 0.761 (95% CI 0.648–0.873), 0.834 (95% CI 0.743–0.925), and 0.855 (95% CI 0.771–0.940), respectively (Fig. 7b). The AUC values further confirmed the accuracy and discriminative ability of the nomogram. The DCA curves in both the training and validation sets demonstrated that compared to the traditional TNM staging, the nomogram model had superior net clinical benefits and could effectively predict the 1-, 2-, and 3-year CSS of patients with MiNENs (Fig. 8).

## Discussion

The large population‐based study allowed us to understand better the incidence, clinical and pathological characteristics, and prognosis of MiNENs. As the largest cancer registration project, the SEER database covers approximately 35% of the US population and is a powerful tool for conducting retrospective population-based studies.

According to our study, the incidence rate of gastrointestinal MiNENs was only 0.227 cases per 1,000,000 person-years in 2000, but by 2019, it had increased by approximately 4.4 times to 1.018 cases per 1,000,000 person-years. Due to the influence of diagnostic and screening methods on tumor diagnosis (Siegel et al. 2023), it was difficult to determine whether the increased incidence rate of MINENs was a genuine rise or was due to improved diagnostic techniques leading to greater clinical identification of MiNENs. In the subgroup analysis of the incidence rate, it was observed that the prevalence of MiNENs was slightly higher in males and elderly patients, consistent with previous studies (Milione et al. 2018; Frizziero et al. 2020). MiNENs had the potential to occur at any location along the digestive tract (Mestier et al. 2017). While data from five European centers indicated that MiNENs most commonly occurred in the colon (Frizziero et al. 2019), other studies suggested that the appendix was the most frequent site of MiNENs (Frizziero et al. 2020; Shi et al. 2020). Our research also found that the incidence of MiNENs was highest in the appendix.

MiNENs and NETs both contained neuroendocrine tumor components, but few studies have directly compared these two tumor types. It remained unclear whether MiNENs and NETs had similar clinical and pathological characteristics. Previous studies have shown that NETs tended to have a higher grade and N stages were mostly N0–1 (Wang et al. 2022), while MiNENs were typically more invasive and poorly differentiated tumors, often associated with extensive lymph node metastasis (Zhang et al. 2021a), which was consistent with the findings of our study.

Until now, most studies on the prognostic differences between NETs and MiNENs were small-scale studies with varying conclusions. Pommergaard et al.’s study found no difference in survival rates between NETs and MiNENs (Pommergaard et al. 2021). Huang et al.’s study found that the median OS of NETs patients was significantly longer than that of MiNENs patients (Huang et al. 2021). In Brathwaite et al.’s study, the total survival period of MiNENs patients was 4.1 years, significantly different from the 13.4 years of adenocarcinoma or NETs patients (Brathwaite et al. 2016). Our study discovered that the median OS of NETs patients was 108 months (102–120 months), significantly longer than the 50 months (36–70 months) observed in MiNENs patients (*P* < 0.001).

Wang et al. discovered that tumors size greater than 2 cm, distant metastasis, and lymph node metastasis were independent risk factors for adverse prognosis in MiNENs patients and were independently associated with increased risk of death (Wang et al. 2020). Our study also obtained consistent conclusions through univariate and multivariate Cox regression analysis. Due to the rarity and heterogeneity of MiNENs, there are currently no clear surgical guidelines or unified treatment standards (Zheng et al. 2020). However, by referring to the treatment protocols for gastrointestinal NETs and adenocarcinoma, surgical resection remains the preferred treatment option for MiNENs, as long as it is feasible (Tanaka et al. 2017). We also observed that local resection and radical resection were protective prognostic factors for MiNENs patients.

Although there have been some studies on MiNENs based on the SEER database, none have created the incidence curves and prognostic nomogram. Shi et al. and Song et al. focused on the characteristics and prognostic factors of MiNENs patients (Song et al. 2022; Shi et al. 2020). Xing et al. concentrated on the therapeutic effects of surgery and postoperative chemotherapy on elderly and non-elderly patients with appendix MiNENs (Xing et al. 2023). In this study, we drew the incidence curves to express the increasing incidence of gastrointestinal MiNENs yearly. In addition, based on independent prognostic factors, an innovative predictive nomogram model was developed to accurately estimate the survival probability of each patient through a user-friendly graphical interface. The accuracy and clinical value of this model were verified, and this nomogram could help doctors make clinical decisions better.

However, our research still had some limitations. Firstly, it was a retrospective study using the SEER database, which may introduce selection bias in the patient selection process. Secondly, the SEER database lacked some vital information, such as tumor composition, pathological morphology, ki-67 index, mitotic count, immunohistochemical markers, metastatic tumor burden, and patient's general condition, which may also be related to the tumor heterogeneity and prognosis of MiNENs patients (Milione et al. 2018; Zhang et al. 2021b). Thirdly, there was a lack of treatment information such as immunotherapy, targeted therapy, and detailed chemotherapy regimens. Fourthly, the lack of information regarding tumor recurrence prevented us from further analyzing the recurrence rate. Fifthly, our study did not have independent external validation. Extensive multicenter clinical studies are needed to validate the accuracy of our model.

## Conclusion

In summary, gastrointestinal MiNENs are rare tumors with an increasing incidence rate and a worse prognosis compared to NETs. Tumor size, distant metastasis, lymph node metastasis, and surgery are independent risk factors for the prognosis of MiNENs patients. A nomogram model has been established based on these factors to predict CSS. It has been validated that the nomogram has the good discriminative ability, consistency, and high clinical utility, which can assist clinical doctors in evaluating individual survival rates and making treatment decisions.

## Data Availability

The datasets used are available from the corresponding author on reasonable request.
